# A Framework for UWB-Based Communication and Location Tracking Systems for Wireless Sensor Networks

**DOI:** 10.3390/s110909045

**Published:** 2011-09-21

**Authors:** Juan Chóliz, Ángela Hernández, Antonio Valdovinos

**Affiliations:** Research Institute of Engineering in Aragón, I3A, University of Zaragoza, C/María de Luna 3, Zaragoza 50018, Spain; E-Mails: anhersol@unizar.es (A.H.); toni@unizar.es (A.V.)

**Keywords:** UWB, wireless sensor networks, location tracking, hybrid positioning and communication, system performance evaluation

## Abstract

Ultra wideband (UWB) radio technology is nowadays one of the most promising technologies for medium-short range communications. It has a wide range of applications including Wireless Sensor Networks (WSN) with simultaneous data transmission and location tracking. The combination of location and data transmission is important in order to increase flexibility and reduce the cost and complexity of the system deployment. In this scenario, accuracy is not the only evaluation criteria, but also the amount of resources associated to the location service, as it has an impact not only on the location capacity of the system but also on the sensor data transmission capacity. Although several studies can be found in the literature addressing UWB-based localization, these studies mainly focus on distance estimation and position calculation algorithms. Practical aspects such as the design of the functional architecture, the procedure for the transmission of the associated information between the different elements of the system, and the need of tracking multiple terminals simultaneously in various application scenarios, are generally omitted. This paper provides a complete system level evaluation of a UWB-based communication and location system for Wireless Sensor Networks, including aspects such as UWB-based ranging, tracking algorithms, latency, target mobility and MAC layer design. With this purpose, a custom simulator has been developed, and results with real UWB equipment are presented too.

## 1. Introduction

The use of location and tracking information is an excellent tool to improve productivity and to optimize resource management in a wide range of sectors: industrial, medical, home-automation or military. Whereas satellite systems, *i.e*., GPS, are widely used in outdoor applications such as vehicle navigation, fleet management or emergency call localization, there are multiple alternatives for the development of indoor Location & Tracking (LT) systems. Despite not being specifically designed for that purpose, various widespread radio technologies such as cellular (GSM, UMTS, LTE) and short-medium range wireless systems (WiFi, Bluetooth, ZigBee, RFID), may provide location information with different levels of accuracy, range and complexity [1,2]. Within this group of short range radio systems, Ultra-Wideband (UWB) stands out providing high accuracy on distance estimation with remarkable features concerning size and power consumption and allowing simultaneous location and data transmission [3].

IR (Impulse Radio) UWB communication systems are based on the transmission of very short duration pulses, which originate very high bandwidth signals. The short duration of the pulses allows a high level of accuracy in time of arrival estimation and as a result a centimeter-level resolution in distance estimation (ranging). Furthermore, due to the short duration of the transmitted pulses, UWB provides unmatched performance on multipath and NLOS environments [4]. In addition, low complexity and low power consumption of UWB transceivers is essential in order to design battery-powered sensors.

In general, location determination comprises two phases, angle and/or distance estimation and position calculation. Angles and distances between the element to be located and some fixed reference nodes can be estimated based on the measurement of different parameters such as Angle of Arrival (AOA), Received Signal Strength Indication (RSSI) and Time of Arrival (TOA) of reference signals exchanged between them. In particular, TOA estimation requires the exchange of ranging frames between the element to be located and the reference nodes, which entails that some temporal resources must be dedicated to location and that a non-negligible latency is associated to the position update process. On the other hand, several algorithms can be used to compute the position according to the estimated distances or angles, including geometry-based (triangulation, trilateration, multidimensional scaling), least square and cost function minimization, fingerprint and Bayesian techniques (Kalman and particle filters).

Extensive research has focused on the design of distance estimation and position calculation algorithms in the last few years [5–8]. Nevertheless, in general these studies focus on algorithm optimization, and simple scenarios with a single terminal and a few previously defined reference nodes are considered. Only a few studies address practical aspects such as the design of the functional architecture, the procedure for the transmission of the associated information between the different elements of the system, and the need of tracking multiple terminals simultaneously in various application scenarios. These aspects would lead to consider the amount of resources associated to the global location service as a quality parameter. Moreover, a rigorous algorithm evaluation requires using a dynamic scenario with multiple mobile terminals and reference nodes.

On the other hand, although a few UWB-based LT systems can already be found in the market, these systems are proprietary solutions and only use UWB for distance estimation, while communication between the different elements involved in the positioning system is done using other technologies, generally wired. In contrast, UWB systems combining location and data transmission (for example in the framework of IEEE 802.15.4a standard) would increase flexibility reducing the cost and complexity of the system deployment.

IEEE 802.15.4 standard offers the fundamental lower network layers of wireless personal area networks (WPAN) focusing on low-cost, low-speed ubiquitous communication between devices with little to no underlying infrastructure [9]. It provides support for *ad hoc* networks capable of performing self-management and organization, aimed at Wireless Sensor Network (WSN) applications. IEEE 802.15.4a-2007 enhanced the standard specifying two additional PHYs: Chirp Spread Spectrum and Direct Sequence UWB, which enhances the standard with the accurate distance estimation capability of UWB [10]. This way, a UWB-based Wireless Sensor Network (e.g., a fire detection sensor network) could be simultaneously used to provide mobile users tracking. In this scenario, it becomes clear that accuracy is not the only evaluation criteria of LT systems, but also the amount of resources associated to the location service, as it has an impact not only on the location capacity of the system, but also on the consequent reduction of the available data rate for sensor data communication.

The combination of wireless sensor networks with UWB accurate location capabilities enables a wide variety of application scenarios. For example, in order to guarantee the safety of workers in dangerous environments (electrical substations, fires, accidents, *etc*.) tracking their position and monitoring at the same time the level of different parameters (electric field, carbon monoxide, radiation, *etc*.). Another example is sports tracking, in order to provide a complete monitoring of performance (distance travelled, average and peak speed, acceleration, *etc*.) and biometric information (heart rate, blood pressure, *etc*.). In industrial applications, the location of a certain product in the assembly line or in a warehouse could be monitored, together with its temperature, humidity, *etc*.

The main objective of this paper is to design a communication and location tracking system for Wireless Sensor Networks based on Ultra-Wideband technology and to provide a complete system-level evaluation. Besides distance estimation and location and tracking algorithms, system level evaluation includes aspects such as target mobility, functional architecture, distribution of the information related to the location function and latency of the position update process. These aspects are not usually considered in the existing studies, but have a great impact on the capacity of systems combining data transmission and location.

The paper is structured as follows. Section 2 summarizes the literature related to the presented study. In Section 3 the proposed communication and location tracking UWB system is described, including the network topology, the tracking functional architecture, the distribution and acquisition of location information and the tracking function implementation. In Section 4 the system performance is evaluated using a custom self-developed simulator, and the different system design alternatives are assessed. Measurements with real UWB equipment are also provided. Section 5 summarizes the main conclusions.

## 2. Related Work

As it was previously mentioned, most of the previous works related to UWB location and tracking systems focus on distance estimation techniques and location and tracking algorithms, but only a few of them address practical aspects such as the transmission of the information associated to location among the different elements of the system. Some proposals of UWB-based communication and location tracking systems can be found in the literature. In [11] a UWB-based system for indoor location services is introduced, which relies on a three-tier hierarchical sensor architecture to cover a large indoor space, also defining the communication between the different tiers and the location procedure. In [12] the design of a UWB-based *ad hoc* network for search and rescue operations in disaster zones is presented, defining the network architecture, physical entities and a complete protocol stack, from the physical layer up to the application layer. In [13] a group of communication protocols and localization algorithms for wireless sensor networks in coal mine environments is proposed, specifically a new UWB coding method, an ALOHA-type channel access method and a message exchange protocol to collect location information. Finally, in [14] an overview of an IR UWB open prototyping platform that illustrates a fully integrated solution from physical layer up to application layer is provided. However, these works [11–14] mainly focus on the system description, including in some cases a very basic system evaluation. Based on the system proposed in [14], this paper presents a thorough analysis and evaluation of different aspects such as the functional architecture, the acquisition and distribution of the information associated to location and the effect of position update latency and target mobility.

The optimization of MAC layer design for simultaneous location and communication has also been addressed in different works. In [15] an early approach to UWB MAC layer issues for location and tracking applications is provided and various UWB system architectures, MAC schemes and network solutions are discussed, although no evaluation of the proposals is provided. In [16] a MAC layer design for IR UWB location networks is proposed in order to determine the locations of a network of stationary reference nodes and mobile nodes deployed in an *ad hoc* manner based on a small number of fixed anchors with known locations. Location and range information is propagated through the network of reference nodes to periodically estimate the locations of the mobile nodes, which entails a convergence time and associated throughput. On the contrary, the tracking system proposed in the present work considers that the locations of the reference nodes are known, whether they are known a priori or determined in a prior set-up phase. This way the convergence time and associated throughput are minimized, thus providing fast tracking of the mobile nodes. The MAC layer design we have considered as the basis for the proposed UWB communication and location tracking system was presented in [17]. This MAC is based on the IEEE 802.15.4 standard [9], although it deviates from the standard in a few areas such as the support to peer-to-peer communications, the usage of guaranteed time slots for data transmissions and dedicated time slots for ranging and allocation requests, the definition of a relaying functionality and the specification of ranging and localization procedures. In [18] the performance of this MAC is studied under the point of view of tracking, evaluating the time delay necessary to collect the ranging information as a function of the number of mobiles in the network. Furthermore, a few enhancements are proposed in order to minimize the exchange of packets necessary to update the ranging information. However, this study assumes the existence of physical connectivity between all the nodes and does not take into account the resource constraints of the MAC layer design, aspects that are considered in this study, which also evaluates the performance degradation in terms of accuracy due to the latency associated to the position update process.

## 3. Wireless Sensor Communication and Location Tracking System Proposal

The proposed communication and location tracking UWB system aims to enable wireless data communication within a network of sensors and at the same time to track walking users in wide indoor areas with accuracy below 1 m. The system is composed of multiple UWB picocells. Each picocell is composed of mobile nodes to be tracked (targets) and fixed sensor nodes with known positions (anchors). Distances between the target and the anchor nodes are estimated through a ranging frame exchange. Estimated distances are sent to location controllers (LC), which are the functional units that execute the tracking algorithm to obtain the estimated position of the targets. The main characteristics of the network and the different options considered are detailed below.

### 3.1. Network Topology and PHY/MAC Structure

The application scenario is covered by multiple UWB picocells, although for simplicity a single picocell is considered. PHY and MAC layers of an open IR-UWB platform described in [14] are assumed. This platform is based on the 802.15.4a standard [10], although it is not fully compliant. Table 1 summarizes the main PHY and MAC parameters considered.

**Figure 1.**
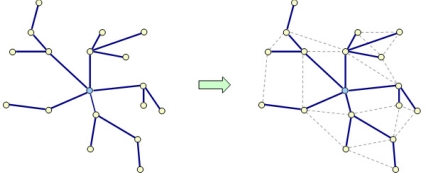
Mesh centralized topology.

The picocell topology is mesh centralized, as shown in Figure 1. A picocell coordinator transmits beacon frames for common superframe synchronization and handles the scheduling procedures. Then, a scheduling tree is built and used to transport beacon and command frames, which are relayed from the picocell coordinator to any node in the picocell. Finally, it becomes a meshed scheduling tree by enabling the transmission out of the tree for the data, ranging and hello frames.

**Figure 2.**
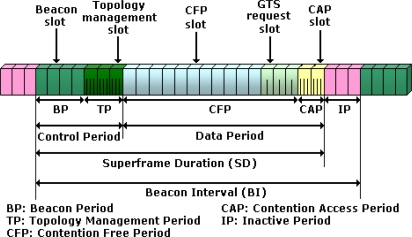
Proposed MAC superframe structure.

The MAC superframe is divided into timeslots that are grouped into different periods, as it is shown in Figure 2.

- Beacon period: Used for the beacon alignment. The first beacon slot is reserved for the coordinator.
- Topology Management Period: Used for the periodic broadcast of hello frames from each node. This way the neighborhood is known locally for each node of the network.
- Contention Free Period (CFP): It is composed of Guaranteed Time Slots (GTS) for sensor data, location data and ranging frames transmission, and a GTS request period. Concerning data frames, if source and destination nodes are not physically connected frames are relayed at MAC level using consecutive timeslots. Ranging frames are not relayed and can be sent only between neighbor nodes. Two types of ranging frames are defined: ranging request and ranging response.
- Contention Access Period (CAP): Used for the transmission of command frames through a slotted ALOHA multiple access scheme. Each CAP slot is divided into subslots in order to relay commands.

It should be noted that the relaying procedure is performed at the MAC layer level. When a node has data to transmit, it sends a GTS request on the tree to the coordinator with its address as the source address and the destination address of the transmission. The coordinator, which has the knowledge of the whole network, looks on its routing table if there are relays between the source and the destination. If there are relays, the coordinator determines the route and allocates the GTS for each link.

### 3.2. Functional Architecture and Strategies for Acquisition and Distribution of Location Information

In order to track the position of the target nodes, location information, basically the distances estimated between the target and the anchor nodes, must be acquired and transmitted to a LC that executes the tracking functionality. LCs can be physically located in one or more anchor nodes or in the target nodes. Depending on the location of the LC, several tracking functional architectures (centralized and distributed) can be defined. On the other hand, either the target or the anchor nodes may estimate the distance. This function is referred to as distance acquisition function. The allocation of the distance acquisition function to the target or the anchor nodes is a design alternative that may have an impact on the need of resources.

#### 3.2.1. Tracking Function Distribution in the Network

**Figure 3.**
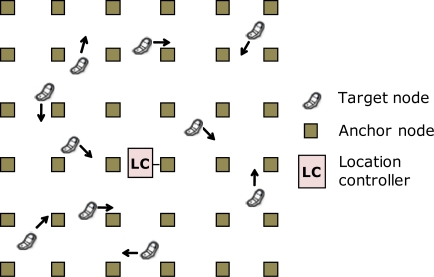
Tracking system. Centralized architecture with 1 LC.

Depending on the location of the LC function, different tracking functional architectures can be defined. In the tracking functional architecture that we denote as centralized architecture, the tracking functionality is implemented in one or more previously defined anchor nodes that become LCs. Figure 3 shows an example of a centralized architecture with one LC. Using one LC entails a higher need of resources, as multiple hops will be needed to forward the location information to the LC. Defining multiple LCs reduces the need of resources, but increases complexity, as the tracking functionality must be implemented in several nodes and a procedure should be implemented to assign each target to the closest LC.

In the distributed architecture, each target dynamically picks one of its neighbor anchors to execute the tracking functionality. Therefore, there may be as many simultaneous LCs as targets. As the LC is always executed by an anchor neighbor to the target, only one timeslot will be needed to exchange data frames between the target and the LC, and resources will consequently be reduced. As a drawback, the tracking functionality must be implemented in every anchor.

Finally, in the target-centered architecture the LC function is implemented in the target nodes. The target nodes perform ranging with their neighbor anchors and obtain their own position applying the tracking algorithm. Therefore, there is no need of transmitting the estimated distances and the updated position. Nevertheless, the implementation of the tracking functionality requires certain computational capacity on the target nodes, increasing their complexity and cost.

#### 3.2.2. Acquisition and Distribution of Location Information

The acquisition of the location information is done through the ranging procedure. The procedure initiator (target or anchor) transmits a ranging request to another node, which estimates the time of arrival and sends a ranging response after a predefined time. The initiator measures the time of arrival of the response and can estimate the transmission delay and the distance between the nodes (Two Way Ranging). In order to improve the accuracy of distance estimation, two ranging responses can be sent in order to compensate for the clock drift (Three Way Ranging).

Once the distances between the target and the anchor nodes have been estimated, they must be transmitted to the LC in a location data frame. The LC calculates and transmits the updated position to the target node. In order to reduce the amount of resources needed to acquire and distribute the location information, different enhancements can be applied [18].

- Data aggregation: All the distances estimated can be aggregated in a single location data frame and sent to the LC by the ranging initiator (target or anchor).
- Broadcast/multicast request: The ranging initiator (target or anchor) can aggregate multiple ranging requests into a single ranging request sent to all its neighbor nodes (broadcast request) or to a subset of them (multicast request).
- Multicast response: After receiving the ranging requests from different nodes, an anchor or target node can aggregate all the responses into a single multicast response.

If the initiator is the target node, multicast response would require the simultaneous update of the position of all the targets, so the anchors can aggregate the responses to multiple targets into a single multicast response packet. The same applies for broadcast/multicast request and data aggregation when the initiator is the anchor node.

### 3.3. Tracking Function Implementation

With respect to the tracking technique itself, parametric and non-parametric approaches can be distinguished. Parametric approaches compute the location based on the a priori knowledge of a model. On the other hand, non-parametric approaches do not require model knowledge, although in some cases they may use some statistic parameters (mean, variance). Specifically, the following algorithms are considered in this study: Trilateration, Least Square-Multidimensional Scaling (LS-MDS), Least Square-Distance Contraction (LS-DC), Extended Kalman Filter (EKF) and Particle Filter (PF).

Trilateration is a non-parametric algorithm that computes the position based on the distance estimated between the target and three anchor nodes using a geometrical method for determining the intersection of three sphere surfaces [7]. Consequently, regardless of the number of anchors selected, only the three anchors with smallest estimated distance to the target are used for position computation.

The algorithm LS-MDS is a completely non-parametric approach combining Multidimensional Scaling (MDS) with Least Squares (LS) minimization [19]. MDS is a multivariate data analysis technique used to map “proximities” into a space. These “proximities” can be either dissimilarities (distance-like quantities) or similarities (inversely related to distances). Given *n* points and corresponding dissimilarity, MDS finds a set of points in a space such that a one-to-one mapping between the original configuration and the reconstructed one exists. Then it is possible to map back the solution to the absolute reference system by Procrustes transformation. MDS is used to obtain a previous estimation of the solution. Then, the localization problem is posed into a non linear least squares optimization problem. The goal is to obtain the matrix of computed positions X that minimizes the stress function σ (*X*) defined as follows:

(1) $$\sigma \left(X\right)={\sum_{i=1}^{n}\sum_{j=1}^{n}\left(\partial_{\mathrm{ij}}-d_{\mathrm{ij}}\left(X\right)\right)}^{2}$$

where ∂*_ij_* is the estimated distance between nodes *i* and *j* and *d_ij_(X)* is the distance between nodes *i* and *j* associated with the computed node locations *X*. In order to solve this problem, a low-complexity algorithm based on majorization technique is applied. Specifically, the algorithm is known as SMACOF and it consists of an iterative procedure that attempts to find the minimum of a non-convex function by tracking the global minima of the so-called majored convex function successively constructed from the original objective and basis on the previous solution.

LS-DC combines the Distance Contraction (DC) algorithm with Least Square minimization [20]. Firstly, the distances between the target and *n* anchor nodes are estimated. Each estimated distance defines a line of position for the target’s location as a circle around the corresponding anchor node. Then, the feasibility region is defined as the area of intersection between the *n* circles. If the feasibility region does not exist, distance contraction cannot be applied and the LS-MDS approach is used instead. If the feasibility region exists, an initial point is computed inside the feasibility region and the contracted distances are computed as the shortest distance from each anchor to the aforementioned feasibility region. Finally, a minimization algorithm is applied using the contracted distances instead of the estimated ones. Since the function becomes convex, any minimization algorithm (*i.e*., global distance continuation, steepest descent) can be used thus reducing complexity, although here SMACOF has been used in order to be comparable with LS-MDS.

The Extended Kalman Filter is a Bayesian technique known for its low complexity, performance and stability as a tracking algorithm [21]. EKF addresses the problem of trying to estimate the state *x* of a discrete-time controlled process that is governed by a non-linear stochastic difference equation:

(2) $$x_{t}=f\left(x_{t-1},u_{t-1},w_{t-1}\right)$$

with a measurement *z* that is:

(3) $$z_{t}=h\left(x_{t},e_{t}\right)$$

The non-linear function *f* in Equation (2) relates the state at the previous time step *t − 1* to the state at the current time step *t* and includes as parameters any driving function *u_t_* and the process noise *w_t_*. The non-linear function *h* in Equation (3) relates the state *x_t_* to the measurement *z_t_* and includes as parameter the measurement noise *e_t_*. Process and measurement noise are assumed to be independent, white, and with normal probability distributions *p(w)* ∈ *N(0, Q)* and *p(e)* ∈ *N(0, R)*. The process and measurement noise covariance matrixes *Q* and *R* are defined by variances *σ_w_^2^* and *σ_e_^2^*.

The Kalman-based tracking algorithm has two major stages, namely, the update and the correction stages, which are iterated *k* times for every observation occurring at a given time. The time update equations project the state and covariance estimates from the previous time step *t − 1* to the current time step *t*. The measurement update equations correct the state and covariance estimates with the measurement *z_t_*. As *f* and *h* cannot be applied to the covariance directly, matrixes of partial derivatives (Jacobian) are computed.

Focusing on the implementation of the EKF for the tracking application, the state vector *x* contains target’s position *p_t_* and speed *v_t_* as process variables. The measure vector *z* contains the process observations, namely the estimated distances between the target and the anchors. The functions that describe the evolution of the state vector through time and the relation between the state vector and the measure vector are:

(4) $$\left(\begin{array}{c} p_{t+1}\\ v_{t+1} \end{array}\right)=\left(\begin{array}{cc} I & T_{s}\cdot I\\ 0 & I \end{array}\right)\;\left(\begin{array}{c} p_{t}\\ v_{t} \end{array}\right)+\left(\begin{array}{c} T_{s}^{2}/2\cdot I\\ T_{s}\cdot I \end{array}\right)w_{t}$$

(5) $${\tilde{z}}_{t}\left(i\right)=\left|p_{i}-{\tilde{p}}_{t}\right|+e_{t}$$

where *T_S_* is the time between two consecutive updates and *p_i_* is the position of anchor *i*.

Finally, Particle Filters are recursive implementations of Monte Carlo based statistical signal processing. The use of particle filters for positioning in wireless networks was proposed in [22]. The particle filter is based on a high number of samples of the state vector or particles, which are weighted according to their importance (likelihood) in order to provide an estimation of the state vector. The advantage of particle filters over other parametric solutions is that non-linear models and non-Gaussian noise can be defined. As a drawback, their computational complexity is higher, so they are suitable in applications where computational power is rather cheap and the sampling rate slow. As for EKF, a state vector *x*, a measure vector *z* and functions *f* and *h* are defined. On each step, the particles are moved according to Equation (4) and the weights are updated according to the likelihood of the observations:

(6) $$w_{t}^{i}=w_{t-1}^{i}p\left(z_{t}|x_{t}^{i}\right),\;\;i=1,\ldots ,N$$

where *i* is the particle index, *N* is the number of particles and the probability $p\left(z_{t}|x_{t}^{i}\right)$ is equivalent to the probability $p_{e}\left(z_{t}-h\left(x_{t}^{i}\right)\right)$ according to the distribution of the measurement error *e*. But here the measurement noise *e* is not necessarily considered Gaussian. Specifically, we have defined the measurement error model as a weighted sum of three Gaussian components for the different channel configurations (LOS/NLOS/NLOS2). The weight of each component is also a Gaussian-like function as will be later defined in Equation (9). Consequently, the filter is defined by the variance of process noise *σ_w_^2^* and the parameters of the measurement error model (mean and variance of each component and mean and variance of each weight).

## 4. Performance Evaluation

### 4.1. System Model and Simulator Description

In order to evaluate the impact of the different system design alternatives and parameters, we have developed a specific simulation application using C++. The simulation scenario represents a relatively wide indoors area, such as a warehouse, where people and goods moving at pedestrian speeds will be tracked with accuracy below 1 m. On this scenario a UWB-based wireless sensor network composed of *N_a_* anchor nodes and *N_m_* target nodes is deployed. The existence of walls and obstacles is considered through the use of an indoor ranging model which accounts for the probability of non-line-of-sight between targets and anchors. The dynamics of the targets are modeled by a Random Walk Model, with random directions and speeds that are constant during a certain period of time, after which new random directions and speeds are set [23].

A common set of parameters has been defined. Area size has been set to 50 m × 50 m in order to represent a relatively wide indoors area. UWB nodes range has been set to 15 m according to the specifications of the IR-UWB platform presented in Section 3.1 [14]. In order to guarantee connectivity between adjacent anchors, the distance between adjacent anchors has been set to 10 m, which results into 36 anchors. Concerning the dynamics of the target nodes, the Random Walk Model is defined by the minimum speed, the maximum speed and the change interval, that have been set to 0.1 m/s, 3 m/s and 20 s respectively in order to model pedestrian motion. Finally, as a result of prior simulations, the nominal position update interval has been set to 976 ms (5 superframes), as it provides accurate tracking of the moving targets with a reasonable use of resources (timeslots).

A ranging model is used to characterize the ranging error distribution and to generate the distance estimation samples. Range measurements based on round-trip TOA estimation through n-Way Ranging transactions can be modeled as:

(7) $${\tilde{d}}_{\mathrm{ij}}=d_{\mathrm{ij}}+{ɛ}_{\mathrm{ij}}+n_{\mathrm{ij}}=d_{\mathrm{ij}}^{\prime }+n_{\mathrm{ij}}$$

where *d_ij_* is the actual distance between nodes *i* and *j*, *d_ij_′* is the biased distance (with bias *ɛ_ij_*) and *n_ij_* is a residual error term.

The biased distance is modeled as a weighted sum of Gaussian and Exponential components conditioned upon the actual distance and channel configuration. The pdf of *d′*, conditioned upon *d* and a particular channel configuration *C*, is described as follows:

(8) $$p_{C}\left[d^{\prime }/\left(d,C\right)\right]=G_{C}\frac{1}{d}\frac{1}{\sqrt{2\pi }\sigma_{C}}e^{-\frac{{\left(\frac{d^{\prime }}{d}-1\right)}^{2}}{2\sigma_{C}^{2}}}+E_{C}\frac{1_{\left\{d^{\prime }>d\right\}}}{d}\lambda_{C}e^{-\lambda_{C}\left(\frac{d^{\prime }}{d}-1\right)}$$

where *d* ≠ 0, {*G_C_*, *σ_C_*} and {*E_C_*, *λ_C_*} are the weights and parameters of Gaussian and Exponential mixture components, 1_{x > y}_ = 1 whenever x > y and 0 otherwise, and *C* takes its value among {LOS, NLOS, NLOS2}. The model is enhanced by taking into account the probability *W_C_(d)* to have a particular channel configuration at a distance *d*. These weights are described as Gaussian-like functions:

(9) $$W_{C}\left(d\right)=\frac{\xi }{\sqrt{2\pi }\zeta_{C}}e^{-\frac{{\left(d-d_{C}\right)}^{2}}{2\zeta_{C}^{2}}}$$

This model for the ranging bias was proposed and validated through a measurement campaign with real UWB equipment in an office environment in [24], where the values for the different parameters of the model were also identified.

The residual error is modeled as additive and centered, with a variance σ*_n_^2^* that depends on detection error terms affecting unitary TOA estimates, *i.e*., receiver sampling rate, and involved protocol durations, and is independent of the distance between the nodes.

**Figure 4.**
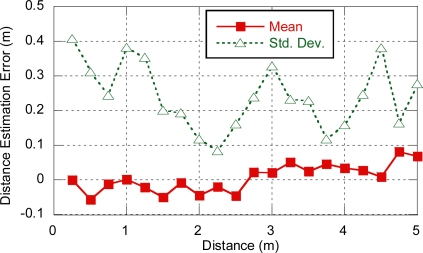
Distance estimation error.

In order to set a realistic value for ranging residual error σ*_n_*, a measurement campaign was carried out using the open IR-UWB platforms mentioned in Section 3.1. Line-of-sight and distances up to 5 m in steps of 0.25 m were considered. Under these conditions, the error is mostly due to residual error. For each distance, 150 ranging samples were obtained and the mean and standard deviation of the distance estimation error was computed. As it can be observed in Figure 4, the mean is always close to zero and no dependency on distance has been detected, as it was expected for ranging residual error. On the other hand, standard deviation varied from 10 cm to 40 cm, with an average value of 25 cm. As a result, we will consider a value of σ*_n_* = 0.3 m for the subsequent simulations.

### 4.2. Simulation Results

In this section, the system performance is evaluated and the different design alternatives are assessed. In Subsection 4.2.1 the performance of the different LT algorithms considered is evaluated using an ideal no-delay approach. The effect of delay on LT algorithms is analyzed in Subsection 4.2.2 considering generic position update latency. In Subsection 4.2.3 the specific MAC structure is taken into account, and the effect of target mobility is discussed. In Subsection 4.2.4 the different proposed strategies for acquisition and distribution of location information are assessed with the common centralized architecture with 1 LC, followed by the assessment of more advanced tracking functional architectures in subsection 4.2.5. The complete system evaluation considering all the different aspects is provided in subsection 4.2.6. Finally, experimental results with UWB prototypes are also provided.

#### 4.2.1. Tracking Algorithms

The performance of each algorithm has been evaluated depending on the number of anchors used for positioning. In a first step, distance estimation and position calculation are ideally considered instantaneous, so the potential error associated with delay is not present. Concerning EKF and PF, prior simulations have been carried out in order to find the optimum values of the process and measurement noise parameters that minimized the error.

**Figure 5.**
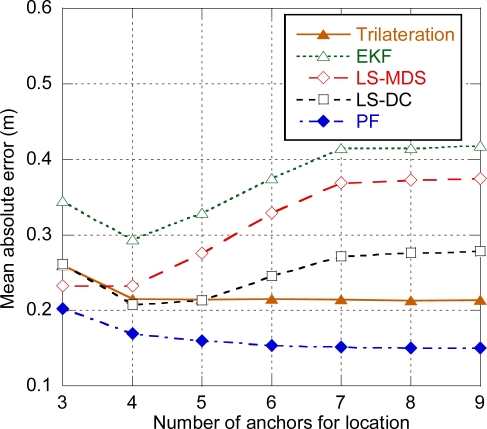
Positioning error. Distance between anchors = 10 m, σ_n_ = 0.3 m.

Figure 5 shows the average positioning error for a ranging residual error *σ_n_* = 0.3 m. As it can be observed, the best performance is achieved with the particle filter. Nevertheless, the performance of the particle filter is far better than it can be expected in a real situation. The reason is that, after being optimized through simulations, the measurement error model of PF behaves almost exactly as the ranging model used to generate the distance estimation samples. Consequently, PF can deal even with highly biased measurements and the error of the particle filter decreases as the number of anchors used for location increases. In a real system, the precise characterization of the specific ranging model of the scenario would require costly measurement and calibration phases, and the use of a generic model would not provide so good results.

Trilateration, LS-MDS and LS-DC show a similar value for minimum error slightly over 20 cm, compared to 30 cm for EKF. The optimum number of anchors is four for LS-DC and EKF and three for LS-MDS. If more anchors are used, the added anchors will be more distant and will have higher ranging bias, thus increasing the positioning error. On the other hand, trilateration is almost independent on the number of anchors used to compute the position, as only the three closest anchors will be used. For every algorithm, there is an increase of the error when only three anchors are used, and the error remains constant for more than seven anchors, as the target is not likely to be in coverage of more than seven anchors.

**Figure 6.**
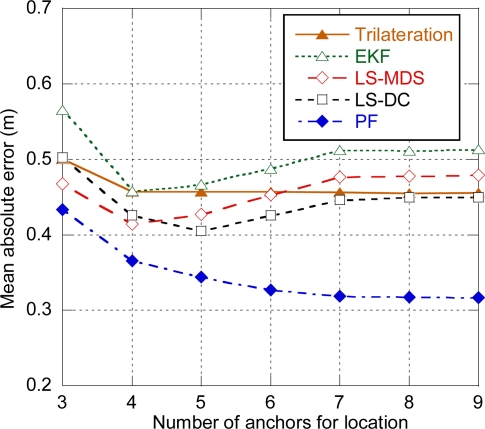
Positioning error. Distance between anchors = 10 m, σ_n_ =0.6 m.

Figure 6 shows the average positioning error for a more pessimistic value of ranging residual error (*σ_n_* = 0.6 m). As expected, the average error is increased compared to the same configuration with ranging residual error *σ_n_* = 0.3 m. The error increase for trilateration is especially remarkable, with results comparable to EKF in terms of minimum error. This means that trilateration requires accurate TOA estimation in order to provide good results, as it always uses three measurements for position computation and cannot take advance of diversity of measurements. Consequently, LS-MDS and LS-DC are preferred over trilateration.

**Figure 7.**
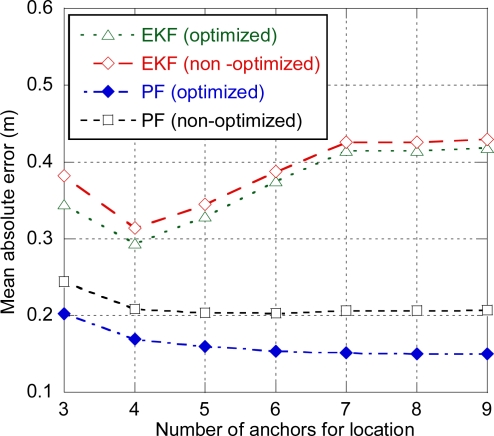
Impact of model optimization in EKF and PF. Distance between anchors = 10 m, σ_n_ = 0.3 m.

As it was previously mentioned, parametric approaches such as EKF and PF require the accurate characterization of the target’s motion model and the measurement model. With this purpose, the optimum values of the different parameters of EKF and PF that minimized the error for certain conditions (scenario layout and ranging error) were obtained through prior simulations. In order to assess the impact of the accuracy of model characterization on the positioning error, the performance of EKF and PF using parameter values that are not optimized for the current conditions is compared to the performance of the optimized filters. Figure 7 shows the average positioning error for both optimized and non-optimized EKF and PF for a ranging residual error *σ_n_* = 0.3 m. Optimized filters use the parameter values resulting of the optimization for *σ_n_* = 0.3 m as in Figure 5. On the other hand, in order to assess the impact of an incorrect calibration, non-optimized filters use parameter values different from the optimum. In case of the non-optimized EKF, instead of using the optimum values (resulting of the optimization for *σ_n_* = 0.3 m), the measurement noise variance *σ_e_^2^* is set to the value resulting of the optimization for *σ_n_* = 0.6 m. That is to say, we use a calibration obtained for different conditions (*σ_n_* = 0.6 m) instead of the calibration obtained for the current conditions (*σ_n_* = 0.3 m). As it can be observed, there is little degradation in the EKF performance, but it should be noted that the optimized EKF showed the worst performance among the different algorithms, as the Gaussian measurement noise assumption is not appropriate to model the ranging error, which is highly biased. Concerning the non-optimized PF, the value of the variance of the LOS component optimized for *σ_n_* = 0.6 m has been used instead of the value optimized for *σ_n_* = 0.3 m, and the mean and variance of the NLOS and NLOS2 components have been multiplied by factors 2 and 4 respectively. As expected, the non-optimized PF shows worse performance than the optimized PF, with a minimum error slightly over 20 cm, which is comparable to the performance achieved with non-parametric approaches such as LS-DC (see Figure 5). It must be noted that the parameters (mean and variance) defining the weights of each component have not been modified, although these values depend on the specific scenario and would introduce additional degradation. Finally, it should be also remarked that the difference between EKF and PF performance is mostly due to the fact that EKF uses a simple Gaussian measurement model whereas in PF we have implemented a 3-component model that is able to deal with the biased ranging error, rather than by the filters themselves. Although more complex models can be implemented by combining multiple EKF filters through multihypothesis tracking and Interacting Multiple Model (IMM) methods, the PF is better suited for the implementation of complex measurement models.

#### 4.2.2. Effect of Position Update Latency

According to the position update process previously described and to the MAC superframe structure shown in Section 3.1, there are many sources of delay in the position update process: delay associated to the request and allocation of free ranging slots, duration of the ranging exchanges, transmission of the estimated distances to the location controller, latency of position computation, transmission of the updated position to the target, *etc*.

**Figure 8.**
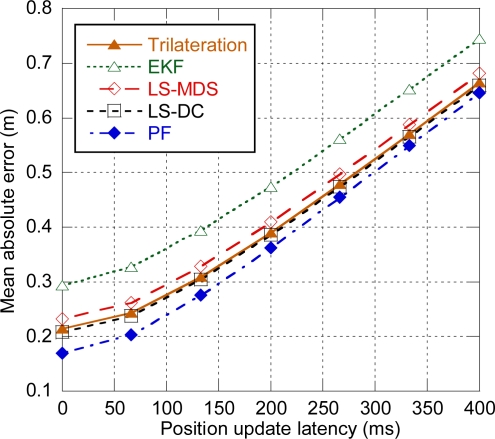
Positioning error depending on position update latency.

Delay prior to the start of the ranging exchanges has no appreciable effect on positioning accuracy. Nevertheless, delay between the ranging exchanges and the final position availability has a negative effect on positioning accuracy due to the movement of the target. We define position update latency as the time between the start of the ranging exchanges and the availability of the position at the target. Figure 8 shows the effect of position update latency on the average positioning error for the different tracking algorithms. As it can be observed, all the algorithms show a similar evolution and the error grows as position update latency increases. For 200 ms that is approximately the duration of a MAC superframe, the average error increases around 18 cm. For 400 ms (two superframes) the error increase can be as high as 45 cm. Therefore, position updates should be carried out preferably within a single superframe.

#### 4.2.3. Effect of Target Mobility

**Figure 9.**
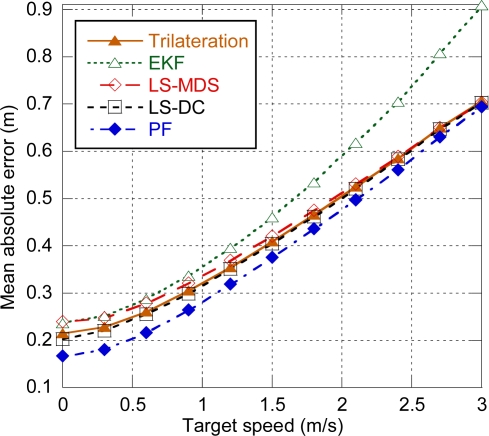
Positioning error depending on target speed.

Next, the effect of target mobility on the positioning error is analyzed, taking into account the timing associated to the MAC superframe structure presented in Section 3.1. Figure 9 shows the positioning error for the centralized architecture with a single LC and four anchors used for location. As it can be observed, when delays are taken into account, all the algorithms are degraded due to the movement of the target between distance estimation and position computation. Non-parametric algorithms, namely trilateration, LS-MDS and LS-DC, which are ideally independent on target speed, have a similar evolution. EKF severely degrades for speeds greater than 1.5 m/s as the estimation is based on target’s previous position. Finally, although PF uses the target’s dynamic model to move the particles, particles are weighted on each step according to their likelihood, so the new position is almost independent of the previous one and degradation is slightly higher than for non-parametric methods.

#### 4.2.4. Impact of Acquisition and Distribution Strategies

**Figure 10.**
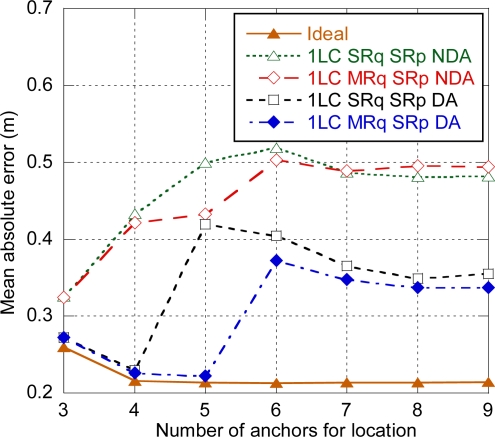
Positioning error for the enhanced modes.

In order to reduce the amount of timeslots needed and consequently the latency, data acquisition and distribution enhancements presented in Section 3.2.2 can be applied. The following notation is used: SRq (Single Request), MRq (Multicast Request), SRp (Single Response), MRp (Multicast Response), NDA (No Data Aggregation), DA (Data Aggregation). The different enhancements have been simulated based on a centralized architecture with one location controller (denoted as 1LC in Figure 10) and one target. Trilateration has been used as location and tracking algorithm as its performance, without considering latency, is almost independent of the number of anchors used for location provided that more than three anchors are used (see Figure 5). This way, when the amount of timeslots needed and consequently the latency linked to data acquisition and distribution is explicitly considered, the impact of each strategy depending on the number of anchors can be appreciated better. As Three-Way ranging is used, two ranging responses are generated for each request, so three slots are needed for each ranging exchange. Concerning estimated distances transmission, in general multiple hops will be needed to relay the data frames from the target to the LC and *vice versa*. As it was specified in Section 3.1, there are 12 ranging slots and eight data slots per superframe.

Figure 10 shows the average positioning error for each one of the acquisition & distribution schemes depending on the number of anchors. The error for an ideal case with no delay is included as a reference. Position update latency and therefore positioning error increase are mainly determined by the number of superframes needed for ranging exchanges and estimated distances transmission. Note that results are constant for seven or more anchors as with this configuration (10 m between anchors) the target is not likely to be in coverage of more than seven anchors. The modes without data aggregation show a similar evolution as they are mainly determined by the number of superframes needed for estimated distances transmission, which depends on the number of anchors used and the number of hops from the target to the location controller. Consequently the error increases as the number of anchors increase. The only difference is for five anchors, as with SRq at least two superframes are needed for ranging exchanges, whereas with MRq ranging exchanges can be completed within one superframe and a second superframe may not be needed if the target is just one hop away from the LC.

When data aggregation is used, position update latency is mainly determined by the ranging exchanges, as location data transmission will be carried out always in a single superframe. With single request (SRq SRp DA), a second superframe will be needed for ranging when five anchors are used, so there is an important increase of the error. Then the error slightly decreases, which is related to the ratio of distances recently estimated (in the second superframe) and delayed (in the first superframe). With multicast request (MRq SRp DA) the error increase occurs when six anchors are used.

**Figure 11.**
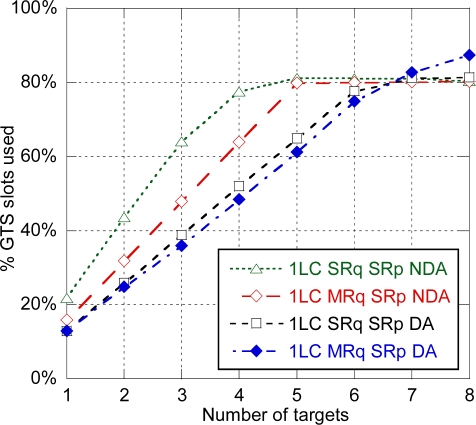
% of GTS slots used for location data transmission for the enhanced modes.

Next the effect of tracking multiple targets simultaneously on system capacity is analyzed. With that purpose, the number of anchors used for location is fixed to four and the number of targets is variable. Figure 11 shows the % of GTS slots used for location data transmission. When no enhancements are applied, the amount of slots used increases quickly and the system is eventually saturated for more than four targets, with a residual 20% left for sensor data communication. The amount of GTS slots used is reduced for the enhanced modes. As with DA a single measurement report packet is sent, capacity of these modes is limited by the availability of ranging slots to five targets (SRq SRp DA), six targets (MRq SRp DA) and eight targets (MRq MRp DA) per picocell.

#### 4.2.5. Impact of the Tracking Functional Architecture

**Figure 12.**
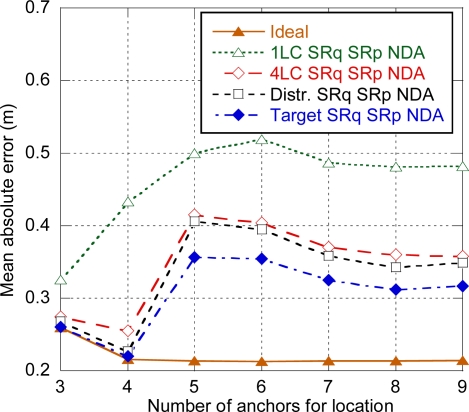
Positioning error for the tracking functional architectures (SRq SRp NDA).

In this section, the different functional architectures of the tracking system that were proposed in Section 3.2.1 are evaluated. Figure 12 shows the average error obtained for the different functional architectures depending on the number of anchors used for location. SRq SRp NDA mode has been considered. Results for the centralized architecture with one LC were already discussed in the previous section. For the distributed architecture, as the LC is implemented in an anchor neighbor to the target, location data transmission can always be done within a superframe, and latency is not determined by location data transmission, but by ranging exchanges. Specifically, as 12 ranging slots are available on each superframe and three slots are required for each ranging exchange in the SRq SRp NDA mode, up to four ranging exchanges can be done in a single superframe, and a second superframe will be needed if more than four anchors are used. Consequently there is an important increase of the error when five anchors are used, and a slight decrease for more anchors, which is again related to the ratio of distances recently estimated (in the second superframe) and delayed (in the first superframe). A similar evolution is shown for a centralized architecture with four LC as most of the time the target will be neighbor to one of the LCs. Finally, the target-centered architecture shows a similar evolution but with a slightly lower error, as there is no need of transmitting the estimated distances and the computed position, so the position update latency will always be a little bit lower.

**Figure 13.**
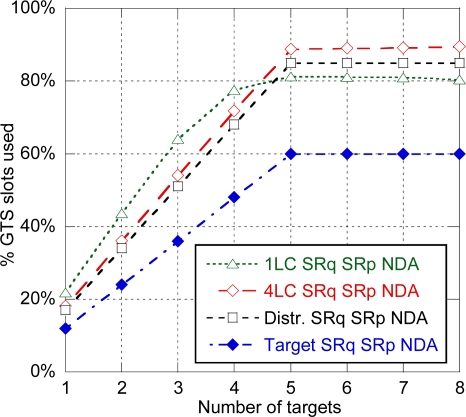
% of GTS slots used for location data transmission for the different architectures (SRq SRp NDA).

Figure 13 shows the % of GTS slots used for location data transmission depending on the number of targets with 4 anchors used for location. As it was previously mentioned, the centralized architecture with one LC can track up to four targets until slots allocated to location data transmission are saturated. The distributed architecture and the centralized architecture with four LCs can track up to five targets until ranging slots are saturated, with a residual 10–15% of slots available for sensor data transmission. Finally the target-centered architecture can also track up to five targets but, as location data transmission is not needed, has a lower use of resources, and consequently higher capacity available for sensor data transmission. Therefore, the target-centered architecture is the optimal in terms of use of resources.

#### 4.2.6. Complete System Evaluation

**Figure 14.**
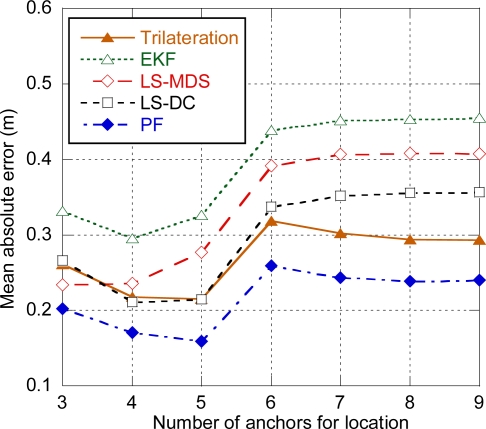
Positioning error for the target-centered architecture (MRq SRp DA).

Finally, accuracy and capacity of the system are evaluated for the different algorithms considering the real MAC implementation. In order to minimize latency, the target-centered architecture centralized has been considered, together with MRq, SRp and DA enhancements. MRq has not been considered as it requires the coordination of the updates, which is complex for the target-centered architecture. Figure 14 shows the average positioning error for the different algorithms. Results for 5 or less anchors are similar than those of Figure 5 as latency is relatively small. When 6 anchors are used, there is an important increase of the error for all the algorithms, as each update will require two superframes thus increasing latency.

**Figure 15.**
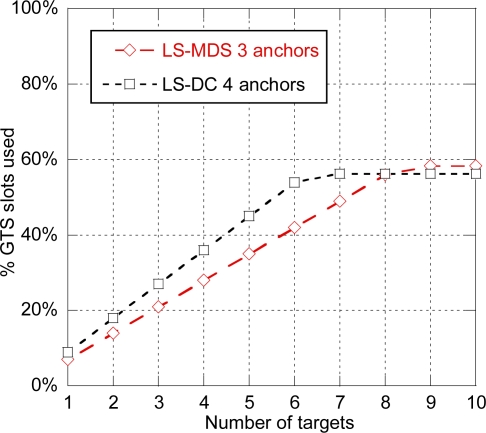
% of GTS slots used for location data transmission for LS-MDS (three anchors) and LS-DC (four anchors).

In order to evaluate capacity, we have selected two options, LS-MDS with three anchors and LS-DC with four anchors, which provide an average positioning error of 23 and 21 cm respectively. Although PF provides better performance, non-parametric approaches such as LS-DC and LS-MDS are preferred over PF as they are completely independent of the scenario and do not require any prior model characterization. Again, the target-centered architecture and MRq, SRp and DA enhancements have been considered. As it can be observed in Figure 15, the system can track up to six anchors in case of LS-DC with four anchors, and up to eight anchors in case of LS-MDS with three anchors, leaving a residual 40% of GTS slots available for sensor data transmission.

#### 4.2.7. Experimental Results

In order to assess the accuracy level that can be reached with real UWB equipment, an experiment was carried out in a room with approximate dimensions of 5 × 3 meters using the open IR-UWB platforms already introduced in Section 3.1. These platforms are prototypes including radio-frequency, baseband and MAC hardware boards and a software MAC running on an FPGA [14]. The modulation scheme is based on Differential Binary Phase Shift Keying (DBPSK), while demodulation is performed using differential correlation between the incoming signal corresponding to the current data symbol and the previous one. The MAC layer is based on IEEE 802.15.4 and has been already explained on Section 3.1, while the main PHY/MAC parameters are shown in Table 1.

**Figure 16.**
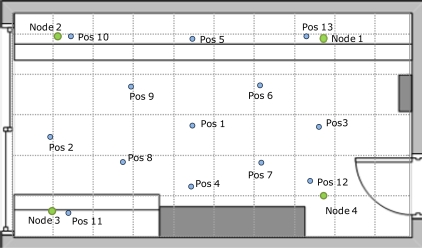
Measurement scenario.

Five prototype devices have been used, one configured as picocell coordinator and anchor node, another one as target node, and the other three as anchor nodes. As the devices are started, the target node estimates the distances to each anchor node and sends them to the picocell coordinator. The picocell coordinator transmits the estimated distances through a serial RS-232 interface. Finally, a computer connected to the picocell coordinator retrieves the distances and computes the position. Consequently, the tracking functional architecture is centralized with one LC, and four anchors are used to locate a single target. LS-DC was implemented as location algorithm as it provides good performance on the different configurations simulated, is independent on target speed and is completely non-parametric, so no prior model characterization is required. Figure 16 shows a plan of the measurement scenario. The anchor nodes (green dots) were placed near the corners of the room and the estimated position was measured in 13 different locations (blue dots).

Table 2 shows the mean and standard deviation of the positioning error on each one of the positions surveyed. The mean absolute error (MAE) varies from 7.8 cm when the target is in the middle of the area (position 1) to 40–45 cm when the target is in a corner of the area (positions 10, 11, 12 and 13). Standard deviation also increases for the corner placements. Average MAE is 26.6 cm, which is slightly higher than the 21 cm obtained for LS-DC in the simulations.

## 5. Conclusions

In this paper, a UWB-based communication and location tracking system for Wireless Sensor Networks has been analyzed. Besides distance estimation and position calculation algorithms, the study covers some other aspects not usually considered on existing studies, such as position update latency, mobility, the functional architecture, the transmission of the associated information, and the need of tracking multiple terminals.

Concerning location and tracking algorithms, the particle filter provides the best results, although measurement model characterization would entail a costly calibration phase, and the use of a generic model would not provide so good results. LS-MDS and LS-DC provide good results and do not require model characterization. Finally, trilateration shows good results for accurate ranging measurements, but degrades as ranging error increases, and the Extended Kalman filter only works well for slow moving targets but severely degrades as target speed increases.

But, as it has been shown, positioning error is highly sensitive to position update latency. Furthermore, when combined data communication and location systems are considered, the use of temporal resources is critical, as it has an impact both on the capacity of the tracking system and the sensor data transmission capacity. In order to deal with latency and resource limitation, different solutions are proposed. The target-centered architecture is optimal in terms of latency and resources needed, as there is no need of transmitting the estimated distances to the network. For any other tracking functional architecture, the use of data aggregation is essential in order to minimize the amount of slots used for location data transmission. Finally, the number of anchors used to locate a certain target should be limited according to the number of slots allocated to ranging, so the position update can be completed within a single superframe.

## Figures and Tables

**Table 1.** PHY and MAC parameters.

**Parameter**	**Value**

Frequency range	3.5–4.5 GHz
Symbol duration	2.88 μs
Raw bit rate	347 kbps
Slot length	160 bytes
Slot duration	3.686 ms
Maximum superframe length	53 slots
Beacon Interval	195.379 ms
Maximum Beacon Period length	12 slots
Max. Topology Management Period length	3 slots (12 subslots)
Maximum CFP length	26 slots
Number of slots for data communication	8 slots
Number of slots for ranging	12 slots
GTS request period length	6 slots (12 subslots)
Maximum CAP length	12 slots (48 subslots)

**Table 2.** Positioning error for the different positions surveyed.

**Positioning error**	**Pos1**	**Pos2**	**Pos3**	**Pos4**	**Pos5**	**Pos6**	**Pos7**	**Pos8**	**Pos9**	**Pos10**	**Pos11**	**Pos12**	**Pos13**
Mean absolute error (cm)	7.8	41.6	29.4	13.6	19.4	14	15.8	17.9	20.5	44.3	44.9	37	40.2
Std deviation (cm)	2.6	4.1	2.8	6.1	2.3	5.3	7.3	3.2	10.9	11	11.8	12.5	5.1
